# Editorial: Microbiome in IBD: From Composition to Therapy

**DOI:** 10.3389/fphar.2021.721992

**Published:** 2021-06-24

**Authors:** Ruixin Zhu, Peijian He, Zhanju Liu, Ningning Liu, Yinglei Miao, Chenggong Yu, Lixin Zhu

**Affiliations:** ^1^ Department of Gastroenterology, The Shanghai Tenth People’s Hospital, Department of Bioinformatics, School of Life Sciences and Technology, Tongji University, Shanghai, China; ^2^ Division of Digestive Diseases, Department of Medicine, Emory University School of Medicine, Atlanta, GA, United States; ^3^ Center for IBD Research, Department of Gastroenterology, The Shanghai Tenth People’s Hospital, Tongji University, Shanghai, China; ^4^ State Key Laboratory of Oncogenes and Related Genes, Center for Single-Cell Omics, School of Public Health, Shanghai Jiao Tong University School of Medicine, Shanghai, China; ^5^ Department of Gastroenterology, First Affiliated Hospital of Kunming Medical University, Yunnan Institute of Digestive Diseases, Kunming, China; ^6^ Department of Gastroenterology, Nanjing Drum Tower Hospital, The Affiliated Hospital of Nanjing University Medical School, Nanjing, China; ^7^ Guangdong Institute of Gastroenterology, Guangdong Provincial Key Laboratory of Colorectal and Pelvic Floor Diseases, Department of Colorectal Surgery, The Sixth Affiliated Hospital, Sun Yat-sen University, Guangzhou, China

**Keywords:** inflammatory bowel disease, microbiome, ulcerative colitis, crohn’s disease, interaction

Inflammatory bowel disease (IBD) is a chronic, non-specific, inflammatory intestinal disease with a high relapse rate. It has become a huge burden worldwide (Kaplan and Ng, 2017). IBD is characterized by changes in the gut microbiome. However, the role of the gut microbiome in the pathogenesis of IBD is still unclear. Therefore, this theme issue aims to discuss the changes of gut microbiome in IBD and its relevance for the treatment of IBD.

Currently, many studies have found that the composition of the gut microbiome in IBD were significantly altered which is considered to contribute to this immune disorder (Glassner et al., 2020). A probable mechanism is that the metabolites of the microbiota regulate metabolic pathways and inflammatory signal transduction thereby affecting the host immune system. On the other hand, the gut microbiota may affect the gut immunity through adherent bacteria. In this issue, Chen et al. reported that in pediatric IBD, adherent bacteria attached to the terminal ileum were associated with elevated Th17 cells and SIgA responses (Chen et al.). The limitation of this study was the lack of intervention approach. Previous studies of adherent bacteria mainly rely on models of immortal cell lines, which cannot reproduce the tissue-like environment. To address limitations, Mayorgas et al. established a human colonic organoids model which represents a promising tool to study the cross talk between Adherent-invasive *Escherichia coli* and intestinal epithelial cells (Mayorgas et al., 2021).

Studies on the microbial change in pharmaceutical treatment of IBD provided additional evidence for the microbial contribution in IBD pathogenesis. Dai et al. investigated the pharmaceutical mechanism of mesalamine in the treatment of UC through 16S sequencing and metabolomics (Dai et al.). Mesalamine is a radical scavenger and antioxidant, often used to treat UC. The authors observed that the abundance of some bacteria and metabolites were reversed toward the healthy status after mesalamine treatment. Similarly, as summarized in this issue by Li et al., many natural products may exert their therapeutic effects on the gut microbiota in the treatment of IBD (Li et al.). These studies reveal possible molecular mechanisms of naturally derived polysaccharides from plants, seaweeds, and mushrooms in the treatment of IBD, and also identified many potential clinical therapeutic targets. In the same direction, Guo et al. reported that ginger can significantly increase the diversity of intestinal microbiome and alleviate the severity of UC (Guo et al.). This study suggests that the abnormality of the microbiome is closely related to amino acid metabolism, oxidative phosphorylation, protein translation, and ribosome biogenesis. Also in this issue, the study of Zhao et al. is unique in utilizing a nano-targeted drug delivery system to achieve a highly efficient delivery of berberine (Zhao et al.), which effectively regulated inflammation and reconstructed the intestinal microbial community.

One surprising report by Liu et al. explored the effect of metformin in the treatment of IBD from the perspective of drug repurposing (Liu et al.). Although metformin is commonly used to treat type 2 diabetes, some studies report that metformin affect the intestinal microbiome and thus may have a beneficial effect on IBD (Sanchez-Rangel and Inzucchi, 2017). Liu et al. demonstrated a positive effect of metformin on IBD and its association with altered microbiota, that is, significantly increased abundance of the anti-inflammatory bacterium *A. muciniphlia* in the metformin treatment group. Interestingly, Silverstri et al. reported that the anti-inflammatory effects of fish oil and cannabidiol are also associated with increased abundance of *A. muciniphlia* in mouse enteritis models (Silvestri et al.). These studies suggest that microbial intervention could be an effective strategy to control inflammation.

Recently, fecal microbiota transplantation (FMT) gains popularity as an intervention to restore the community of the intestinal microbiota and to treat several gastrointestinal diseases (Weingarden and Vaughn, 2017), including IBD, which was reviewed by Tan et al. in this issue (Tan et al.). This review provided an overview of the effectiveness of FMT in the treatment of IBD. Further, the authors summarized the therapeutic mechanisms of FMT in IBD.

The research topic of “Microbiome in IBD: From Composition to Therapy” discuss not only the role of intestinal microbiome in the pathological mechanisms of IBD, but also in the pharmaceutical mechanisms of multiple drugs. We expect that this collection of inspiring work will stimulate new avenues of thinking, approaches, and collaborations in exploring the complex pathogenesis of IBD and lead to novel therapeutic targets for IBD.
